# Development and evaluation of an online questionnaire to identify women at high and low risk of developing gestational diabetes mellitus

**DOI:** 10.1186/s12884-022-04629-8

**Published:** 2022-04-14

**Authors:** Daria Di Filippo, Chloe Bell, Melissa Han Yiin Chang, Justine Darling, Amanda Henry, Alec Welsh

**Affiliations:** 1 School of Women’s and Children’s Health, University of New South Wales, Sydney, NSW Australia; 2 Diabetes Clinic, Royal Hospital for Women, Sydney, NSW Australia; 3 Department of Maternal-Fetal Medicine, Royal Hospital for Women, Locked Bag 2000, Barker Street, Randwick, NSW 2031 Australia

**Keywords:** Gestational diabetes mellitus, Questionnaire, Oral glucose tolerance test, Risk factors, Screening, continuous glucose monitoring

## Abstract

**Background:**

Established risk factors for Gestational Diabetes Mellitus (GDM) include age, ethnicity, family history of diabetes and previous GDM. Additional significant influences have recently been demonstrated in the literature. The oral glucose tolerance test (OGTT) used for GDM diagnosis has sub-optimal sensitivity and specificity, thus often results in GDM misdiagnoses. Comprehensive screening of risk factors may allow more targeted monitoring and more accurate diagnoses, preventing the devastating consequences of untreated or misdiagnosed GDM. We aimed to develop a comprehensive online questionnaire of GDM risk factors and triangulate it with the OGTT and continuous glucose monitoring (CGM) parameters to better evaluate GDM risk and diagnosis.

**Methods:**

Pregnant women participating in two studies on the use of CGM for GDM were invited to complete the online questionnaire. A risk score, based on published literature, was calculated for each participant response and compared with the OGTT result. A total risk score (TRS) was then calculated as a normalised sum of all risk factors. Triangulation of OGTT, TRS and CGM score of variability (CGMSV) was analysed to expand evaluation of OGTT results.

**Results:**

Fifty one women completed the questionnaire; 29 were identified as ‘high-risk’ for GDM. High-risk ethnic background (*p* < 0.01), advanced age, a family diabetic history (*p* < 0.05) were associated with a positive OGTT result. The triangulation analysis (*n* = 45) revealed six (13%) *probable* misdiagnoses (both TRS and CGMSV discordant with OGTT), consisting of one *probable* false positive and five *probable* false negative by OGTT results.

**Conclusions:**

This study identified pregnant women at high risk of developing GDM based on an extended evaluation of risk factors. Triangulation of TRS, OGTT and CGMSV suggested potential misdiagnoses of the OGTT. Future studies to explore the correlation between TRS, CGMSV and pregnancy outcomes as well as additional GDM pregnancy biomarkers and outcomes to efficiently evaluate OGTT results are needed.

**Supplementary Information:**

The online version contains supplementary material available at 10.1186/s12884-022-04629-8.

## Background

Gestational diabetes mellitus (GDM), defined as hyperglycaemia during pregnancy that is not diabetes, currently affects approximately 14% of Australian pregnancies [1, 2]. On top of the known risk factors for GDM (non-Caucasian ethnicity, high body mass index -BMI, older maternal age, family history and previous diagnosis of GDM or polycystic ovary syndrome – PCOS) additional factors have been proposed to impact GDM risk and confirmed in systematic reviews recently [3–12]. They include physical exercise, the use of assisted reproductive technologies and cholesterol and iron intake [13–19].

The pregnant body undergoes significant physiological changes to support the growing demands of the fetus. A uniform 50–60% decrease in insulin sensitivity throughout gestation is described in the literature [20, 21]. In GDM, an alteration of the insulin receptors combined with underlying subclinical factors (as metabolic syndrome/high BMI/PCOS) accentuates the insulin resistance, resulting in beta-cell dysfunction and maternal hyperglycaemia [21, 22].

The GDM hyperglycaemic intrauterine environment stimulates fetal hyperinsulinemia and fetal growth, resulting in obstetric, perinatal and long-term complications [23–26]. The risk of developing type 2 diabetes mellitus (T2DM) is reported to be seven times higher for women affected by GDM and their infants have almost double the risk of developing childhood obesity [21, 27]. This highlights a venomous intergenerational cycle of obesity and diabetes influencing the health of the entire population.

Within the past decade, the incidence of GDM has doubled, making it the fastest-growing subtype of diabetes in Australia [2]. This is due to advancing maternal age, an increased prevalence of maternal obesity and a change in diagnostic criteria [2, 28]. Australia currently proposes universal testing for GDM, regardless of pre-screening risk, using a 75 g oral glucose tolerance test (OGTT) at 24–28 weeks’ gestation [29]. Women having one or more risk factors for GDM are tested the earliest possible in pregnancy and, if negative, retested at 24–28 weeks’ gestation with no other measurement or preventative measure in place [29].

A comprehensive evaluation of GDM risk factors could allow more targeted prevention, detection, and management in patients at high risk of developing GDM. Our primary aim was to develop and evaluate the use of an online questionnaire on well-established and emerging risk factors confirmed in systematic reviews or studies with at least 500 participants to identify pregnant women at high and low risk of developing GDM. The secondary aim was to evaluate the acceptability and feasibility of the questionnaire for the patients. Thirdly, we evaluated the correlation between the total risk for GDM, the OGTT results, and the variability of blood glucose assessed with Continuous Glucose Monitoring (CGM).

## Methods

### Study design

In this prospective cohort study, participants were invited to participate in an online questionnaire, requiring approximately 15 min to complete. Following completion of the primary survey, participants were re-directed to a secondary survey to evaluate the feasibility, accessibility and acceptability of the survey. Sample size was calculated by considering (a) likely ability of risk factor questionnaire to differentiate between high-risk and low-risk women, with assumption made that high-risk women on TRS would have a 30% chance of having GDM, while low-risk on questionnaire women would only have a 5% chance of having GDM (b) likely proportion of women enrolling who would be high-risk versus low-risk (scenarios run for 1:1, 1:3 and 1:4 enrolment). For 80% power and alpha of 0.05, required sample size to detect questionnaire discriminating ability of 30% GDM for high-risk versus 5% for low-risk varied from 70 to 96 women in total [30].

### Population

Population Pregnant women enrolled in two pilot studies on the use of CGM for the diagnosis of GDM, one completed using the Medtronic iPro2 (Medtronic, Northbridge, CA) [31] and one ongoing using the Freestyle Libre Pro (Abbott, Chicago, IL) [32], were recruited. The inclusion criteria were hence those of the two pilot studies (women between 12 and 35 weeks recently diagnosed with GDM at their routine OGTT, both before and after their first GDM education, or those willing to undergo OGTT during CGM monitoring for the Medtronic pilot study and women between 12 and 29 weeks recently diagnosed with GDM at their routine OGTT, before their first GDM education, or those willing to undergo OGTT during CGM monitoring for the Abbott pilot study). GDM was diagnosed with a 75 g OGTT based on the IADPSG criteria [33]. Participating women were originally recruited for the pilot studies in person or via phone while receiving antenatal care in one of two metropolitan Sydney hospitals and were re-contacted through email, message and phone call to participate to our study. Participants were excluded if they were under 18 years of age, had been diagnosed with diabetes pre-pregnancy, had a psychiatric illness that precluded informed consent or a poor understanding of English that jeopardised informed consent.

### Questionnaire design

#### Primary questionnaire

The questionnaire was administered through the Research Electronic Data Capture (REDCap) platform [34]. An online signature was used to obtain consent. OGTT results were obtained through the eMaternity database. Questions on well-established risk factors such as ethnicity, BMI, medical history (obstetric inclusive) and family history were included, as well as more recently proposed risk factors such as exercise and dietary patterns, season of conception and ART use. Each alternative response was allocated a value based on the odds ratio (OR) for likelihood of development of GDM, as reported in recent studies performed in at least 500 patients when a metanalysis was not available (Supplementary file 1). The baseline risk was considered as being 1 for each risk factor in absence of it, i.e. 1 = baseline, risk factor not present. For detailed examples of the risk factors evaluation, see Supplementary file 1.

Pre-pregnancy BMI was inserted by women if known or calculated from pre-pregnancy height and weight which were requested as well. Pre-pregnancy exercise patterns were determined through a series of questions providing examples of activities (Supplementary file 2). Average duration of each physical activity per week was calculated (Supplementary file 3). For average daily step count, women were asked to choose between four options (0–3159 / 3160–6318 / > 6.318) [14].

Food intake was measured through a semi-quantitative food-frequency questionnaire (FFQ), asking participants to record their food intake the year prior to becoming pregnant. Participants were also asked to record whether their diet changed drastically after finding out they were pregnant. Answers were arranged into nine categories, ranging from ‘never, or less than once per month’ to ‘6+ per day’, with a standard portion size specified for each food. Intakes of individual nutrients, including heme iron and cholesterol, were calculated by multiplying the frequency of consumption of each food by their known average nutrient content, based on food composition data from the U.S. Department of Agriculture (Supplementary file 4) [35].

#### Secondary questionnaire

The secondary survey comprised four questions regarding the accessibility, acceptability and understandability of the questionnaire, recorded along a Likert scale of 0–5. A final free text box allowed participants to share any recommendation or comment (Supplementary file 2).

### Baseline statistical analysis

Statistical analysis was performed using SPSS version 23 (SPSS Inc., Chicago, IL). Continuous variables are presented as mean ± standard deviation when normally distributed, and as median [interquartile range] when non-normally distributed. Comparison of continuous variables between GDM and NGT groups was by independent samples t-test for normally distributed data and Mann-Whitney-U test for non-normally distributed data. Categorical variables are reported as number (percentage) and were compared between groups using chi-square or Fisher’s exact test. Associations between each risk factor and subsequent GDM diagnosis were explored by comparing the proportions (using Chi-squared or Fisher’s exact test) of GDM vs NGT patients with each risk factor*.* Values of *p* < 0.05 were considered statistically significant.

### Risk score evaluation and triangulation

The list of risk factors for GDM considered within the South-Eastern Local Health District (SESLHD), where this study took place, are outlined in Supplementary material 5 [36].

A total risk score (TRS), derived from our questionnaire, was calculated through the sum of the values recorded for each answer then normalised by dividing each individual TRS by the highest TRS recorded. The TRS cut-off value was achieved by finding the midpoint of the highest TRS in the NGT women and the lowest TRS in the GDM women.

A CGM score of variability (CGMSV) was calculated based on three days in a row of monitoring. The parameters considered were of distribution of the glucose levels (mean, SD, coefficient of variation), variability parameters (MAGE - Mean Amplitude of Glycaemic Excursion for intra-day variability, and MODD - Mean of Daily Differences for inter-day variability) and the percentage of time spent in/ above/ below the range recommended for pregnant women (3.5–7.8 mmol/L) [37, 38]. These values were calculated manually after downloading raw data from the CGM systems. MAGE was calculated using the Easy GV software [39]. MODD was calculated manually as “mean of paired blood glucose values on successive days” to allow for the reduced number of values given by the most recent CGM Freestyle Libre PRO compared to the old generation CGM used when the software was created [38].

CGMSV was calculated as a sum of the normalised values of mean, SD, CV, MAGE, MODD, TBR, TAR. The cut-off for CGMSV was determined through the same principle as the TRS cut-off (finding the midpoint of the highest CGMSV in the NGT women and the lowest CGMSV in the GDM women). Triangulation was achieved through comparison of OGTT results, TRS and CGMSV and was interpreted for potential misdiagnosis of the current gold standard, the OGTT. Misdiagnosis were considered *probable* when TRS and CGMSV were both concordant against OGTT: *probable* false negative if OGTT negative but TRS and CGMSV above the cut-offs and *probable* false positive if OGTT positive but TRS and CGMSV below the cut-offs (i.e. true and false positives and negatives are defined with reference to the current “gold standard” of OGTT).

## Results

### Recruitment

**Fig. 1:**
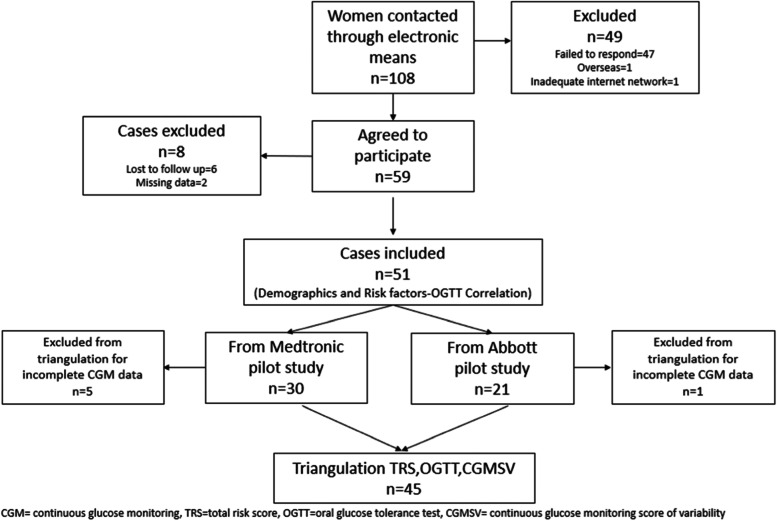
Consort diagram

A total of 108 women were contacted and asked to complete the questionnaire from June to September 2021. Fifty-nine women agreed to participate, with 6 lost to follow up and 2 having incomplete data sets. Of the 51 women recruited, 21 wore the Abbott Freestyle Libre device and 30 wore the Medtronic device. Twenty-nine of the recruited patients were classified as having high risk of GDM. One patient from the Abbott pilot study and five from the Medtronic pilot study couldn’t be included in the triangulation analysis as they had incomplete CGM data (Fig. 1).

### Demographic characteristics

Maternal demographic characteristics are summarised in Table 1. A significantly higher proportion of GDM women were from a high-risk background (47%) compared to NGT women (6%) (*p* < 0.01). Mean BMI was higher in the GDM population, although not significantly (23.5 versus 22.2, *p* = 0.11). Age was higher in NGT women (33.7 versus 31.1, *p* = 0.03).

**Table 1:** Demographic statistics of participants

	GDM (*n* = 15) n (%)	NGT (*n* = 36) n (%)	*p*-value
High Risk Background*	7 (47)	2 (6)	**< 0.01**
Previous GDM	2 (13)	1 (3)	0.14
Family History of DM	8 (53)	7 (19)	**0.02**
	**Mean ± SD**	**Mean ± SD**	
Age	31.1 ± 3.0	33.7 ± 4.0	**0.03**
	**Median (IR)**	**Median (IR)**	
BMI	23.5 (10.4)	22.2 (6.75)	0.11
Parity	0.0 (1)	0.0 (1)	0.78

*GDM* Gestational diabetes mellitus, *NGT* normal glucose tolerance, *DM* Diabetes Mellitus, *SD* Standard deviation, *IR* interquartile range, *BMI* Body Mass Index

*High risk background = Southeast Asian, Chinese, Middle Eastern, Hispanic, South American, Aboriginal, Torres Strait Islander

### Risk factor association

Tables 2 and 3 detail the association between GDM diagnosis and all inherent and acquired risk factors. When subdivided into specific ethnicity groups, the only significant association was seen for the Chinese population (*p* = 0.04). Similarly, a paternal family history of T2DM was significantly associated with GDM risk (33% vs 6%, *p* < 0.01).

**Table 2:** Risk factors and GDM association

	GDM (*n* = 15) n (%)	NGT (*n* = 36) n (%)	*p*-value
Chinese	3 (20)	1 (3)	**0.04**
Southeast Asian	1 (7)	0 (0)	0.12
Middle Eastern	0 (0)	0 (0)	N/A
ATSI	0 (0)	0 (0)	N/A
Paternal T2DM/H	5 (33)	2 (6)	**0.01**
Maternal T2DM/H	1 (7)	3 (8)	0.84
Sibling GDM/H	0 (0)	0 (0)	N/A
Previous GDM	2 (13)	1 (3)	0.14
Previous Macrosomia	0 (0)	1 (3)	0.51
1st TM GWG > 3.78 kg	2 (13)	6 (17)	0.77
PCOS	4 (27)	8 (22)	0.73
Use of ART	0 (0)	2 (6)	0.35
Conception in winter	1 (7)	5 (14)	0.47

*ATSI* Aboriginal or Torres Strait Islander, *T2DM/H* Type 2 diabetes mellitus history, *GDM/H* GDM history, *1st TM GWG* First trimester weight gain, *ART* assisted reproductive technique

**Table 3:** Pre-pregnancy dietary and exercise patterns

	GDM (*n* = 15) Median (IR)	NGT (*n* = 36) Median (IR)	*p*-value
Average daily cholesterol consumption (mg)	162.45 (136.34)	153.40 (136.35)	0.82
Average serving of red meat/ day	0.49 (0.36)	0.38 (0.34)	0.57
Average serving of processed meat/ day	0.13 (0.49)	0.13 (0.26)	0.99
Average Iron intake/ day (mg)	2.78 (2.02)	2.07 (2.21)	0.52
Pre-pregnancy exercise (avg minutes/ week)	166.15 (267.27)	192.89 (219.11)	0.66
	**n (%)**	**n (%)**	
Pre-pregnancy Egg consumption, > 7/ week	2 (13)	4 (11)	0.82
Pre-pregnancy daily avg. step count > 6400	5 (33)	16 (44)	0.46

*Avg* average

No statistically significant difference was seen between the average daily cholesterol, iron intake and step count, despite the higher means for each category in GDM women (Table 3).

### TRS and CGM parameters

Table 4 illustrates the differences in terms of TRS and CGM parameters between women classified as NGT and GDM included in the triangulation analysis (*n* = 45). TRS and CGMSV were significantly higher in GDM compared to NGT women (0.91 vs 0.77 *p* = 0.01 and 4.34 vs 3.66 *p* = 0.03 respectively). Among the CGM parameters, Mean, TAR, SD, and MODD resulted higher in GDM women.

**Table 4:** TRS and CGM parameters

	GDM (*n* = 14) Median (IR)	NGT (*n* = 31) Median (IR)	*p*-value
TRS	0.91 (0.14)	0.77 (0.11)	**0.01**
CGMSV	4.32 (1.36)	3.66 (0.91)	**0.03**
Mean	5.22 (1.17)	4.38 (0.81)	**< 0.01**
TBR	0.47 (0.58)	0.28 (0.39)	0.275
TAR	0.09 (0.9)	0.00 (0.09)	**0.01**
	**Mean ± SD**	**Mean ± SD**	
SD	0.97 ± 0.19	0.85 ± 0.17	**0.04**
CV	0.18 ± 0.05	0.19 ± 0.05	0.66
MAGE	2.40 ± 0.54	2.12 ± 0.51	0.10
MODD	1.10 ± 0.25	0.86 + 0.20	**< 0.01**

*TRS* Total risk factors score, *CGMSV* Continuous glucose monitoring score of variability, *TBR* Time below range, *TAR* Time above range, *SD* Standard deviation, *CV* Coefficient variation, *MAGE* Mean amplitude of glycaemic excursion, *MODD* Mean of daily differences

### Acceptability and feasibility of primary questionnaire

There was minimal trouble accessing the questionnaire, with 55% of participants rating 5/5 for accessibility. Thirty-seven women (73%) rated 5/5 for understanding all the questions asked, with a further 9 women (18%) rating it 4/5. Additionally, 63% of participants rated the survey as 5/5 for acceptability. Thirty-six women (71%) rated the questionnaire a 4/5 or above as a recommended form of GDM screening. Within the free text section, the majority of comments were positive. Seven women (14%) mentioned that they had trouble remembering their pre-pregnancy habits. Three women made comments on the small amount of food allocation options and a further 5 women made comments on the questionnaire display on a mobile device.

### Total risk score, CGMV and OGTT triangulation

The maximum TRS in the NGT population was 0.93 and the minimum score in the GDM population was 0.68. Therefore, the cut off value was determined to be 0.80; anyone above this value was considered to be at high-risk of GDM.

Similarly, the maximum CGMSV in the NGT population was 4.94 and the minimum score in the GDM population was 2.72. Therefore, the cut off value for CGMSV was determined to be 3.83.

**Fig. 2:**
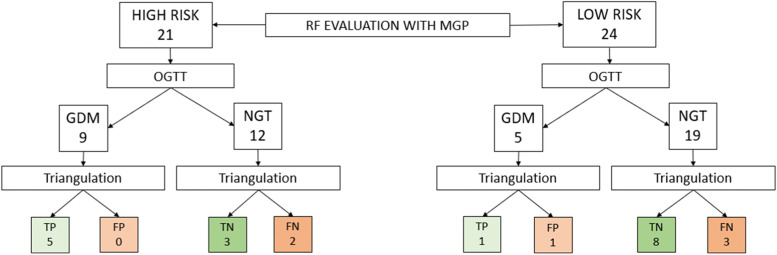
OGTT, TRS and CGMV triangulation.

Triangulation between TRS, CGMSV and OGTT results was performed on 45 women with complete CGM data (Fig. 2). Of the 21/45 (47%) women considered at high risk using the local policy, nine (42%) were positive to the OGTT, of whom five (55%) had TRS and CGMSV above the cut-off (true positive diagnosis) and none had both TRS and CGMSV below the cut-off (*probable* false positive diagnosis). Of the remaining 12 women considered at high risk but with negative OGTT, three (25%) had TRS and CGMSV below the cut-off (true negative diagnosis) and two had both TRS and CGMSV above the cut-off (*probable* false negative diagnosis). Of the 24/45 (53%) women considered at low risk as per the local policy, five (21%) were positive to the OGTT. Among these, one had both TRS and CGMSV above the cut-off (true positive diagnosis), one had both TRS and CGMSV below the cut-off (*probable* false positive diagnosis). The remaining 19 women, considered to be at low risk (79%) of developing GDM, had low TRS and CGMSV in eight cases (true negative diagnosis) and high TRS and CGMSV in three cases (*probable* false negative diagnosis). The remaining patients had OGTT concordant with either TRS or CGMSV, as fully described in Supplementary file 6.

## Discussion

As the incidence of high BMI and advancing maternal age continues to increase, the prevalence of GDM continues to rise [2]. This phenomenon places the mother and fetus at risk of GDM consequences, kickstarting the intergenerational cycle of obesity and diabetes. In this study, we created an online self-administered questionnaire that tested for the presence of both well established and more recently proposed risk factors for GDM. To the best of our knowledge, this has not been previously undertaken. Additionally, our study proposes the triangulation of extensive risk factors assessment with OGTT results and CGM score of variability in an attempt to identify OGTT misdiagnoses.

### Questionnaire design and implications

The use of an online platform (REDcap) to project the survey, allowed us to collect substantial amounts of data efficiently (brief collection time and no risk of errors for data transcription) and economically (low human resources needed) [40, 41]. In a study by Kongsved et al. 97.8% of participants who completed an online questionnaire did so without missing data, compared to only 63.4% completeness using paper-based questionnaires [42]. In our study only 2 women (3% of initially recruited), failed to complete the entirety of the questionnaire.

A Likert scale was chosen to ensure an accessible and adequate display of alternative responses while data remained suitable for quantitative analysis [43]. Most women found the questionnaire accessible and easy to understand, as per the secondary survey results.

Several factors and biomarkers, as well as combinations of those, are reported in literature to be linked with the development of GDM [44–46]. Previous attempts of creating self-administered questionnaire for GDM screening showed good potential, although still requiring some level of interaction with the electronic medical record and only assessing well established risk factors [47–49].

### Risk factors and GDM in our cohort

In our analysis, we confirmed a significant association between OGTT and well-established risk factors such as family history of diabetes and high-risk ethnic background. In contrast with what described in literature, NGT women of our cohort were older than GDM women [6]. When analysed, the correlation between increasing BMI and older age is higher in the GDM group than the NGT group, although this difference is not significant. This could be explained by the higher socioeconomic status and healthier diet of the older women in our cohort.

We found a paternal history of diabetes to be the only statistically significant result when broken down into specific subgroups of family history, as consistent with a recent study [8]. A meta-analysis and systematic review by Bosdou et al., found a significant association between the use of ART and increased risk of GDM, possibly correlated to the use of progesterone to support the luteal phase [17, 50]. We were unable to confirm this in this study, which may be due to our relatively small sample size.

A recent systematic review on women’s pre-pregnancy diet patterns showed that egg intake > 7 per week, cholesterol intake > 300 mg per day, iron intake > 1.1 mg per day and an increase in serving from one red meat and processed meat per day, all placed women at risk of GDM [18]. We found no statistically significant difference for any of these factors between the GDM and NGT populations. A reason for inconsistency may be due to the fact that we only asked about 8 food items (Supplementary file 4) among the several nutrients containing cholesterol and iron. The number of questions in an online survey greatly influences patient response reliability and to reduce the risk of careless responding, it was essential that the survey was not too long [51]. Other studies investigating pre-pregnancy diets on GDM risk have used a 133-items FFQ, finding significant associations between increased pre-pregnancy potato, fried-food, and sugar-sweetened beverage consumption and GDM risk [52–54]. We deemed it implausible for the women to fill a 133-items FFQ out in addition to the other questions. Extending the number of options in our questionnaire might be well-tolerated by patients, as there were comments in the free text section about the lack of food options. The inclusion of such factors would make the questionnaire more sensitive and reliable.

### OGTT limits and results’ interpretation

The OGTT has been greatly contested since 1954 in literature, ten years before its application to the diagnosis of GDM, with concerns that several factors may influence results including timing of samples, diet, exercise, age, gastrointestinal factors and stress [55, 56]. More recently, the OGTT has been reported as unpleasant, expensive, time-consuming, and having poor reproducibility [55, 57–59]. The development of a CGM-based diagnostic test for GDM could represent a solution to the OGTT limits. Many patients are unable to attend a laboratory for three hours because working, taking care of other children or living remotely with no access to transports. The coronavirus 2019 pandemic (COVID-19) has then brought to light new negative implications of the OGTT for pregnant women and the need for a new diagnostic test. Travel restrictions and the need to spend up to three hours in a potentially infectious environment contribute to the barriers women now face if wishing to complete their OGTT [60]. In the hospital where this study took place fasting blood glucose ≥5.1 mmol/ L or HBA1C ≥41 mmol/mol (5.9%) were introduced to diagnose GDM instead of OGTT. McIntyre et al. addresses the changes countries have made to enable women to be tested for GDM during the pandemic [60]. While the changes created a safer environment for women, detection rates of GDM were reported to be much lower (25% in Australia), raising the possibility of missed GDM diagnoses [60]. The reliability of the current gold standard for GDM diagnosis has, however, been questioned for a long time and the need for a more accurate, reliable, and sensitive test for GDM is becoming clearer.

Within different countries, and different organisations in each country, there is still a lack of consensus on the thresholds to adopt [61, 62]. In Australia, while the IADPSG criteria endorsed by the World Health Organisation in 2013 were adopted by The Royal Australian and New Zealand College of Obstetricians and Gynaecologists shortly after, The *Royal Australian College of General Practitioners* still recommends the use of the 1991 ADIPS criteria [63].

OGTT evaluates a single time in a pregnant woman’s metabolism and fails to account for the everyday diet. Women who are conventionally healthy (eating a balanced diet and having a normal weight) will maintain consistent blood sugar levels throughout pregnancy yet may potentially react with an abnormal temporary spike to the ingestion of a supraphysiological carbohydrate load. Consequently, they can be labelled as having GDM, with resulting increased surveillance during pregnancy (‘false positive’ diagnoses). Conversely, women who typically consume a diet high in carbohydrates can pass the OGTT, with failure of intervention and subsequent exposure of mother and fetus to the adverse consequences of GDM (‘false negative’ diagnoses).

### CGM and GDM diagnosis

There is limited literature on the use of CGM for the diagnosis of GDM [64–66]. One small study in a sub-Saharan African population compared home glycaemic profiles (using CGM) with OGTT results, concluding that those with a positive OGTT had home glycaemic profiles that were not significantly different to those who tested negative for the OGTT [65]. Other studies have explored the use of CGM for GDM management rather than diagnosis [67–70]. A recent review concluded that the use of CGM may help understanding glycaemic patterns and that the glycaemic variability within a day and within consecutive days can predict pregnancy outcomes [38]. Glycaemic variability at CGM is defined by multiple parameters, of which we considered those most recommended for women in pregnancy [38].

### Total risk score, CGMSV and OGTT triangulation

TRS, CGMSV and all the CGM variability parameters resulted higher in GDM than NGT women, of which only TBR, CV and MAGE non-significantly.

Twenty-eight of the 51 women in our cohort were identified as ‘high-risk’ for GDM. For these women, the use of education classes or online resources during early pregnancy may have aided in modification of behaviours influencing their likelihood of developing GDM.

Triangulation of TRS, CGMSV and OGTT results allowed for a multiparametric evaluation of OGTT validity in 45 women. Given the extensive literature on the pitfalls of the OGTT, a first step towards overcoming this diagnostic test could be represented by putting in discussion its results by analysing their correlation with other indicators of GDM. Although not directly evaluated against clearly diagnostic pregnancy outcomes, CGMSV and TRS are both derived from a combination of factors that have been clearly linked with GDM (risk factors, glucose variability and time in range). We hence consider it plausible to define a *probable* misdiagnosis when OGTT is discordant with both TRS and CGMSV. Triangulation suggested a concerning rate of discordance, with one *probable* false negative diagnosis and five *probable* false positive diagnosis of the OGTT (discordance between OGTT and both TRS and CGM). Three of the probable false negative women were considered at low risk with standard local criteria, then had high risk for GDM with our questionnaire. A false-negative OGTT diagnosis for these women could lead to failure of medical surveillance, placing the mother and fetus at risk of complications. Assessment of an expanded risk factor list for GDM could therefore identify women at higher risk and may highlight the need for an alternative to the OGTT. The evaluation of pregnancy outcomes related directly to CGMSV and TRS could represent the next step to achieve more reliability and further evaluate the OGTT results. Apart from the parallel pilot studies on CGM for diagnosis of GDM, from which the patients were recruited for this work, this is the first study, to our knowledge, to use risk factor demographics and CGM variability to question OGTT validity. Given the previously mentioned limitations of the OGTT and the reported reliability of CGM in giving insight into glucose patterns and predicting pregnancy outcomes as well as its acceptability for pregnant women, CGM could be an appropriate substitute from the OGTT.

### Strengths, limitations and future directions

We created the first screening questionnaire to include both well-known and recently proposed risk factors for GDM. This allowed for a broader assessment of risk factors in a quick, safe and cost-effective manner, compared to other studies using prediction models for GDM including laboratory analysis hence requesting women to attend medical centres. Triangulation with TRS and CGMSV also identified potential OGTT diagnoses as misdiagnosis. As OGTT is still considered the gold standard for GDM diagnosis, it is difficult to categorically prove this hypothesis. Our group recently published a systematic review on the diagnostic indicators of GDM showing that numerous biomarkers may differentiate GDM from normoglycaemic pregnancy [71]. Future studies are needed to explore pregnancy indicators and outcomes of GDM to add other measurable parameters against OGTT validity. Ultimately, a primary health intervention of screening women based on their GDM risk and a more sensitive diagnostic method will decrease the risk of adverse outcomes and over medicalisation. These measures have the potential to reduce maternal stress, negative outcomes for mothers and newborns, as well as diminish the economic burden of wasted health resources.

The major limitations of this study are the modest sample size and the self-reported nature of data, including BMI, age and the presence of risk factors. Due to the restrictions related to the pandemic, we could not recruit women directly from the antenatal care clinics and had to recruit women already participating in two pilot studies. This deeply impacted our possibility to reach the number of participants expected with our sample size calculation. Submitting the questionnaire to all the women during their antenatal visits will allow for a bigger and more representative sample. Some questions may have been seen as overly personal and women may have given misinformation. The questionnaire could be formatted in a manner more compatible with mobile technology to enable women to complete the questionnaire at a time that is most convenient for them. Whilst recruitment was held in two metropolitan hospitals, only two women were recruited from one of these, resulting in a catchment population regarded as of high socioeconomic status and health conscious. This predisposes the study to sampling bias. The possibility of volunteer bias should also be considered as the participants were a compliant subgroup of a volunteer population and may be unrepresentative of the generality of women screened for GDM. A sampling bias should be considered as well given the low response rate among women approached (56%) and will be explored in future work.

The inconvenience of the OGTT has been widely described in literature and almost all the participants in our study, regardless of the presence/absence of risk factors for GDM, were enthusiastic to join the study given the possibility to help eliminating the need to undergo OGTT for pregnant women.

Furthermore, there were no participating women who identified as Indigenous Australians. Considering that Indigenous women are 1.5 times more likely to develop GDM, their inclusion will provide invaluable insight into the acceptability of the questionnaire screening tool and CGM as a diagnostic tool [4].

## Conclusions

Considering risk factors for GDM recently described as significant in addition to the well-established ones, allowed us to refine the risk of developing this condition. Early identification of ‘at-risk’ women allows closer monitoring and more accurate GDM diagnosis. Combined with CGM variability, our widespread consideration of the most significant risk factors can aid in better identifying GDM, in the attempt to overcome the pitfalls of the OGTT. Reducing potential GDM misdiagnosis means reducing the risk of overtreatment as well as the devastating consequences of untreated GDM.

Our questionnaire demonstrated good accessibility and acceptability for participants. In light of the findings of this study, TRS can be further refined to better assess the risk of developing GDM and evaluate OGTT results.

The COVID-19 pandemic accentuated the need for remote screening and for a diagnostic test for GDM that reduces duration of or need for healthcare exposure. CGM has the potential to represent a solution to the OGTT pitfalls. Further research is needed to fully develop a CGM diagnostic test for GDM without relying on the OGTT only.

## Supplementary Information

**Additional file 1.** Supplementary file 1.

**Additional file 2.** Supplementary file 2.

**Additional file 3.** Supplementary file 3.

**Additional file 4.** Supplementary file 4.

**Additional file 5.** Supplementary file 5.

**Additional file 6.** Supplementary file 6.

## Data Availability

The data that support the findings of this study are not publicly available due to their containing information that could compromise the privacy of research participants but are available from the corresponding author in a de-identified manner upon reasonable request.

## Abbreviations

ART: Assisted Reproductive Technology
BMI: Body Mass Index
CGM: Continuous Glucose Monitoring
CGMSV: Continuous Glucose Monitoring Score of Variability
GDM: Gestational Diabetes Mellitus
GWG: Gestational Weight Gain
IADPSG: International Association of the Diabetes in Pregnancy Study Groups
MAGE: Mean Amplitude of Glycaemic Excursions
MPG: Management of GDM policy
MODD: Mean of Daily Differences
NGT: Normal Glucose Tolerance
OGTT: Oral Glucose Tolerance Test
PCOS: Polycystic Ovary Syndrome
SD: Standard Deviation
T2DM: Type 2 Diabetes Mellitus
WHO: World Health Organization
