# Supplementary material for: Development and evaluation of an online questionnaire to identify women at high and low risk of developing gestational diabetes mellitus
Source: BMC Pregnancy Childbirth. 2022 Apr 14;22:321. doi: 10.1186/s12884-022-04629-8 (PMC9009497; doi:10.1186/s12884-022-04629-8)
Supplement: Supplementary file 1 — Additional file 1. Supplementary file 1. [file 12884_2022_4629_MOESM1_ESM.docx]

Supplementary file 1. Calculated OR for Individual Risk Factors

| **Risk Factor** | **Reference**  **Study type (n)*** | **If** | **OR** | **If** | **OR** |
| --- | --- | --- | --- | --- | --- |
| **Ethnicity^3-4^** | ^3^COH (> 5 k) | Southeast Asian^3^ | 3.03 | Middle Eastern | 2.47^3^ |
|  | ^4^SR/MA (>32 mln) | Chinese^3^ | 4.77 | ATSI | 1.42^4^ |
| **BMI^5^** | SR/MA (> 962 k) | Overall population | +0.14 for each unit from 18 |  |  |
| **Age^6^** | SR/MA (>1 mln) | Overall Population | +0.079 for each year >18 | Asian | +0.1274 for each year >18 |
| **Family Hx^7-10^** | SR/MA (>31 k) | Yes^7^ | 3.46 | No^7^ | 1 |
|  |  | Paternal-T2DM | 3.49^8 [COH (912)]*^ | Sibling GDM | 5^9^ [COH (858)]* |
|  |  | Maternal-T2DM | 2.32^8 [COH (912)]*^ |  |  |
| **GDM Hx^10^** | COH (>73 k) | Yes | 5.24 | No | 1 |
| **PCOS Diagnosis^11^** | COH (> 9 mln) | Yes | 2.19 | No | 1.0 |
| **Previous Macrosomia^12^** | C/C (>47 k) | Yes (>4.5kg) | 6 | No | 1 |
| **GWG in First Trimester^13^** C/C (>1 k) | | <3.78kg | 1 | 3.24-5.60kg | 1.43 |
|  |  | >5.61kg | 1.74 |  |  |
| **Steps/day^14^** COH (>700) | | 0 steps | 1 | 0-3159 | 0.81 |
|  |  | 3160-6318 | 0.62 | >9477 | 0.43 |
| **Physical Activity^15^**** COH (>20k) | | <30 | 1 | >210 | 0.78 |
| **Season of Conception^16^** COH (60 k) | | Winter | 1.1 | Spring | 1.042 |
|  |  | Summer | 1 | Autumn | 1.022 |
| **Pregnancy Establishment^17^** SR/MA (>1 mln) | | Spontaneous | 1 | Use of ART | 1.53 |
|  |  | IVF | 1.95 | ICSI | 1.42 |
| **Diet^18-19^**  ^18^ SR (>87 k)  ^19^COH (>13 k) | | >7eggs/wk | 2.65 | >300 mg cholesterol/day | 1.45 |
|  |  | Every 0.5 mg increase from 1.1 mg heme iron/day | 1.22 | Red meat: total increase serving/day  Red meat: increase serving of processed/day | 1.66  1.47 |

COH= cohort, SR= systematic review, MA= meta-analysis, C/C= case control, MLN=million, K=thousand

OR= odds ratio, Hx= history, T2DM=type 2 diabetes mellitus, GDM= gestational diabetes mellitus, BMI= body mass index, ATSI= Aboriginal or Torres Strait Islander, PCOS= polycystic ovary syndrome, GWG= gestational weight gain, ART= assisted reproductive technique, IVF= in vitro fertilization, ICSI= intra cytoplasmic sperm injection,

* (n): number of participants

**Physical activity: minutes per week

Note: the references have the same numeration of the manuscript
