# Supplementary material for: Development and evaluation of an online questionnaire to identify women at high and low risk of developing gestational diabetes mellitus
Source: BMC Pregnancy Childbirth. 2022 Apr 14;22:321. doi: 10.1186/s12884-022-04629-8 (PMC9009497; doi:10.1186/s12884-022-04629-8)
Supplement: Supplementary file 2 — Additional file 2. Supplementary file 2. [file 12884_2022_4629_MOESM2_ESM.pdf]

questionnaire

Development and Evaluation of an Online Questionnaire to identify Patients at High and Low Risk of Developing Gestational Diabetes Mellitus (GDM)

Invitation

You are invited to participate in a research study into the development of an online questionnaire to identify pregnant women at high or low risk of developing gestational diabetes mellitus (diabetes developed during pregnancy). The study is being conducted at the Royal Hospital for Women and St George Hospital by:

•Prof. Alec Welsh, Professor of Maternal Fetal Medicine, Royal Hospital for Women and University of New South Wales

•Dr Amanda Henry, Senior Lecturer in Obstetrics and Gynaecology, Senior Research Fellow, University of New South Wales, Royal Hospital for Women, St George Hospital, and The George Institute for Global Health.

•Dr Daria Di Filippo, PhD Candidate, School of Women's and Children's Health, University of New South Wales

•Dr Melissa Chang, Program Coordinator, School of Women's and Children's Health, University of New South Wales

Before you decide whether or not you wish to participate in this study, it is important for you to understand why the research is being done and what it will involve. Please take the time to read the following information carefully and discuss it with others if you wish.

What is the purpose of this study?

The purpose is to develop and evaluate an online screening questionnaireas a new screening method to help identify those at higher or lower risk of developing GDM.This study forms part of Dr Di Filippo's PhD research and a fourth year medical student's Honours project.

Why have I been invited to participate in this study?

You are eligible to participate in this study because you are pregnant and a patient at the Royal Hospital for Women or St George Hospital, and have either undergone or will be undergoing the Oral Glucose Tolerance Test (OGTT).

What does participation in this study involve?

If you agree to participate in this study, you will be asked to sign the Participant Consent Form, as an e-signature at the bottom of this page.You will be given information on the study and instructions, including the website address of the online questionnaires you will be asked to complete. If you prefer, we can provide a paper version of the questionnaire to fill out. We ask that you submit the completed questionnaires within two weeks of receiving the instructions. The GDM Risk Questionnaire includes questions to evaluate your risk of developing GDM, the second questionnaire will determine your regard of the GDM Risk Questionnaire.By signing the consent form attached you are also giving us permission to access your medical records to collect the relevant study data such as obstetric history and follow up pregnancy outcome. Any information obtained in connection with this research project that can identify you will remain confidential.

What if I don't want to take part in this study, or if I want to withdraw later?

Participation in this study is voluntary. It is completely up to you whether or not you participate. If you decide not to participate, it will not affect the treatment you receive now or in the future. Whatever your decision, it will not affect your relationship with the staff caring for you.

If you wish to withdraw from the study once it has started, you can do so at any time without having to give a reason. However, it may not be possible to withdraw your submitted questionnaire(s) or your data from the study results if these have already had your identifying details removed.

How is this study being paid for?

There is no external funding required to support this study. The time of the researchers is unpaid and forms part of the higher research degree (PhD) work of Dr Di Filippo and research project of an Honours student.

Are there risks to me in taking part in this study?

The only foreseeable risk in taking part in this study is the inconvenience of the time taken to complete the questionnaire(s).

You may feel that some of the questions we ask are stressful or upsetting. If you do not wish to answer a question, you may skip it and go to the next question, or you may stop immediately. If you become upset or distressed as a result of your participation in the research project, the research team will be able to arrange for counselling or other appropriate support. Any counselling or support will be provided by qualified staff who are not members of the research team. This will be provided free of charge.

What happens if I suffer injury or complications as a result of the study?

As participation in this study only involves completing questionnaires in your own time, injury or complications is not anticipated.

Will I benefit from the study?

This study aims to further medical knowledge and may improve future treatment of Gestational Diabetes Mellitus, however it will not directly benefit you.

Will taking part in this study cost me anything, and will I be paid?

Participation in this study will not cost you anything, nor will you be paid.

How will my confidentiality be protected?

Any identifiable information that is collected about you in connection with this study will remain confidential and will be disclosed only with your permission, or except as required by law. Only the researchers named above and necessary others e.g. the Honours Student involved in this project and medical staff involved in your care will have access to your details and results that will be held securely at the Royal Hospital for Women and then securely destroyed 5 years after the last publication.

What happens with the results?

If you give us your permission by signing the consent document, we plan to discuss/publish the results at conferences or other professional forums, and publish in peer reviewed journals and student theses. In any publication, information will be provided in such a way that you cannot be identified.

What should I do if I want to discuss this study further before I decide?

When you have read this information, the researcher will discuss it with you and any queries you may have. If you would like to know more at any stage, please do not hesitate to contact Professor Alec Welsh via email alec.welsh@unsw.eduor by calling (02) 9382 6213.

Who should I contact if I have concerns about the conduct of this study?

This study has been approved by the South Eastern Sydney Local Health District Human Research Ethics Committee. Any person with concerns or complaints about the conduct of this study should contact the Research Support Officewhich is nominated to receive complaints from research participants. You should contact them on 02 9382 3587, or email SESLHD-RSO@health.nsw.gov.au and quote 2020/ETH02618.

Consent

I, (name) agree to participate in the study described in the participant information statement set out above.

2. I acknowledge that I have read the participant information statement, which explains why I have been selected, the aims of the study and the nature and the possible risks of the investigation, and the statement has been explained to me to my satisfaction.

3. Before signing this consent form, I have been given the opportunity of asking any questions relating to any possible physical and mental harm I might suffer as a result of my participation and I have received satisfactory answers.

4. I understand that I can withdraw from the study at any time without prejudice to my relationship to the University of New South Wales Sydney and the Royal Hospital for Women or St George Hospital.

5. I agree that research data gathered from the results of the study may be published, provided that I cannot be identified.

6. I understand that if I have any questions relating to my participation in this research, I may contact Professor Alec Welsh by email alec.welsh@unsw.edu or on telephone029382 6098, who will be happy to answer them.

7. I acknowledge receipt of a copy of this Consent Form and the Participant Information Statement.

After you have read all the above information and agree to participate, please complete the e-signature below.

This questionnaire will take approximately 20 minutes to complete.

____

Development and Evaluation of an Online Questionnaire to identify Patients at High and Low Risk of Developing Gestational Diabetes Mellitus (GDM)

How old are you? ______________________________

Do you have a family history of type 2 diabetes and/ or GDM? ○ No ○ Yes

If yes, what is their relation to you? (check all that apply)

☐ Cousin/ distant relative ☐ Mother-T2DM ☐ Father- T2DM ☐ Sibling ☐ Mother- GDM

Have you previously been diagnosed with GDM? ○ No ○ Yes

Have you previously given birth to a baby weighing >4.5kg? ○ No ○ Yes

Do you identify as Aboriginal or Torres Straight Islander? ○ No ○ Yes

Are you from any of the following backgrounds: Chinese, Southeast Asian, Middle Eastern or Indian? ○ No ○ Yes

If yes, where do you identify as?

○ Hispanic ○ South American ○ Middle Eastern ○ South Asian ○ Chinese

What was your pre-pregnancy BMI?

○ unknown ○ < 18.5 ○ 18.5-24.9 ○ 25-29.9 ○ >29.9

How much weight did you gain in your first trimester? (up to 14 weeks gestation)

○ Not sure ○ < 3.78kg ○ 3.24-5.60kg ○ >5.61kg

Have you been diagnosed with polycystic ovary disease (PCOS)? ○ No ○ Yes

Did you require the use of assisted reproductive technologies (IVF, ICSI) or was the pregnancy spontaneous? ○ No ○ Yes

If yes, what type did you use?

○ Intracytoplasmic Sperm Injection (ICSI) ○ In-Vitro Fertilisation (IVF)

Do you currently, or have you previously taken anti-depressant medication? ○ No ○ Yes

If yes, do/ did you take either of the following two medications? ○ No ○ Venlafaxine ○ Amitriptyline

If yes, have you taken more than two antidepressant medications at once? ○ No ○ Yes

What season did you conceive in?

◯ Winter ◯ Spring ◯ Summer
◯ Autumn

Did you participate in any of the following activities on a regular basis during the year before you became pregnant? (check all that apply)

- ☐ Walking
- ☐ Swimming
- ☐ Jogging
- ☐ Weightlifting
- ☐ Dance/aerobics
- ☐ Bicycling
- ☐ Hiking
- ☐ Yoga
- ☐ Other activity

For walking, how many times per week? ______________________________

For walking, how many months did you regularly participate in this activity? ______________________________

For walking, how much time did you spend at the activity per episode? (in minutes) ______________________________

For swimming, how many times per week? ______________________________

For swimming, how many months did you regularly participate in this activity? ______________________________

For swimming, how much time did you spend at the activity per episode? (in minutes) ______________________________

For jogging, how many times per week? ______________________________

For jogging, how many months did you regularly participate in this activity? ______________________________

For jogging, how much time did you spend at the activity per episode? (in minutes) ______________________________

For weightlifting, how many times per week? ______________________________

For weightlifting, how many months did you regularly participate in this activity? ______________________________

For weightlifting, how much time did you spend at the activity per episode? (in minutes) ______________________________

For dance or aerobics, how many times per week? ______________________________

For dance or aerobics, how many months did you regularly participate in this activity? ______________________________

For dance or aerobics, how much time did you spend at the activity per episode? (in minutes)	________________________
For bicycling, how many times per week?	________________________
For bicycling, how many months did you regularly participate in this activity?	________________________
For bicycling, how much time did you spend at the activity per episode? (in minutes)	________________________
For hiking, how many times per week?	________________________
For hiking, how many months did you regularly participate in this activity?	________________________
For hiking, how much time did you spend at the activity per episode? (in minutes)	________________________
For yoga, how many times per week?	________________________
For yoga, how many months did you regularly participate in this activity?	________________________
For yoga, how much time did you spend at the activity per episode? (in minutes)	________________________
For your other activity, how many times per week?	________________________
For your other activity, how many months did you regularly participate in this activity?	________________________
For your other activity, how much time did you spend at the activity per episode? (in minutes)	________________________

The following questions will ask about your dietary patterns leading up to your pregnancy. For each food listed, choose the circle indicating how often on average you consumed it BEFORE becoming pregnant.

	Never, or less than once per month	1-3 per month	1 per week	2-4 per week	5-6 per week	1 per day	2-3 per day	4-5 per day	6+ per day
Eggs (1)	○	○	○	○	○	○	○	○	○
Beef (1 serving, ~170g)	○	○	○	○	○	○	○	○	○
Lamb (1 serving, ~170g)	○	○	○	○	○	○	○	○	○
Pork (1 serving, ~170g)	○	○	○	○	○	○	○	○	○
Bacon (2 slices)	○	○	○	○	○	○	○	○	○
Processed meats, e.g. salami, sausages, bologna (1 serving)	○	○	○	○	○	○	○	○	○
Shellfish, e.g. shrimp, lobster (1 serving)	○	○	○	○	○	○	○	○	○
Scallops, oysters, clams, mussels (1 serving)	○	○	○	○	○	○	○	○	○

Did you change your diet after finding out you were pregnant/ during pregnancy? ○ No ○ Yes

If you answered yes to the previous question, please re-fill the circles out reflecting how much you consume the listed foods NOW.

	Never, or less than once per month	1-3 per month	1 per week	2-4 per week	5-6 per week	1 per day	2-3 per day	4-5 per day	6+ per day
Eggs (1)	○	○	○	○	○	○	○	○	○
Beef (1 serving, ~170g)	○	○	○	○	○	○	○	○	○
Lamb (1 serving, ~170g)	○	○	○	○	○	○	○	○	○
Pork (1 serving, ~170g)	○	○	○	○	○	○	○	○	○
Bacon (2 slices)	○	○	○	○	○	○	○	○	○
Processed meats, e.g. sausages, salami, bologna (1 serving)	○	○	○	○	○	○	○	○	○
Shellfish, e.g. crab, loyster, prawns (1 serving)	○	○	○	○	○	○	○	○	○
Scallops, oysters, clams, mussels (1 serving)	○	○	○	○	○	○	○	○	○

Thank you for taking the time to complete the main questionnaire. Please rate your experience with this survey and provide any comments that you may have, all comments are appreciated and accepted.

Did you have trouble accessing the questionnaire?

I had great difficulty accessing this questionnaire — The questionnaire was easy to access and I had no trouble

(Place a mark on the scale above)

Did you understand every question in this questionnaire?

I did not understand any question in this questionnaire — I understood every question in this questionnaire

(Place a mark on the scale above)

Did you find this questionnaire generally acceptable?

I found this questionnaire very unacceptable — I found this questionnaire very acceptable

(Place a mark on the scale above)

Would you recommend this form of screening for gestational diabetes to other pregnant women?

I would not recommend — I would recommend

(Place a mark on the scale above)

Do you have any additional comments that you would like to make? All suggestions and comments are welcome. Thank you.

____
