# Supplementary material for: Development and evaluation of an online questionnaire to identify women at high and low risk of developing gestational diabetes mellitus
Source: BMC Pregnancy Childbirth. 2022 Apr 14;22:321. doi: 10.1186/s12884-022-04629-8 (PMC9009497; doi:10.1186/s12884-022-04629-8)
Supplement: Supplementary file 3 — Additional file 3. Supplementary file 3. [file 12884_2022_4629_MOESM3_ESM.docx]

### Supplementary file 3. Pre-pregnancy exercise patterns and Equation

A: “Which activities did you participate in on a regular basis during the year before you became pregnant?”, and for each activity reported:

B: “How many times per week?”

C: “How many months did you regularly participate in this activity?” D: “How much time did you spend at the activity per episode?”

𝐵𝐷(4𝐶) /52
