# Supplementary material for: Development and evaluation of an online questionnaire to identify women at high and low risk of developing gestational diabetes mellitus
Source: BMC Pregnancy Childbirth. 2022 Apr 14;22:321. doi: 10.1186/s12884-022-04629-8 (PMC9009497; doi:10.1186/s12884-022-04629-8)
Supplement: Supplementary file 4 — Additional file 4. Supplementary file 4. [file 12884_2022_4629_MOESM4_ESM.docx]

Supplementary file 4. Nutrient breakdown

| **Food** | **Iron** | **Cholesterol** |
| --- | --- | --- |
| Beef (170g) | 3.75mg | 84mg |
| Lamb (170g) | 4.22mg | 144mg |
| Pork (170g) | 2.92mg | 164mg |
| Bacon (2 rashes) | 1.04mg | 58mg |
| Eggs (1) | 0.88mg | 186mg |
| Sausage (1) | 0.56mg | 49mg |
| Bologna (1 slice) | 0.41mg | 30mg |
| Salami (1 slice) | 1.55mg | 90mg |
| Crab (85g) | 2.45mg | 60mg |
| Lobster (170g) | 2.40mg | 152mg |
| Oyster (1 medium) | 2.56mg | 25mg |
| Mussels (1 cup) | 5.92mg | 42mg |

Based on food composition data from the U.S. Department of Agriculture^35^
