# Supplementary material for: Development and evaluation of an online questionnaire to identify women at high and low risk of developing gestational diabetes mellitus
Source: BMC Pregnancy Childbirth. 2022 Apr 14;22:321. doi: 10.1186/s12884-022-04629-8 (PMC9009497; doi:10.1186/s12884-022-04629-8)
Supplement: Supplementary file 5 — Additional file 5. Supplementary file 5. [file 12884_2022_4629_MOESM5_ESM.docx]

Supplementary file 5. SESLHD Management of Gestational Diabetes Mellitus (GDM) Policy

• Ethnicity: Aboriginal/Torres Strait Islander, Asian, South Asian, Pacific Islander, Maori, Middle Eastern, non-white African

• Insulin resistance (e.g. associated with PCOS)

• Maternal age ≥40 years

• Medications e.g. corticosteroids, antipsychotics

• Periconceptual or initial booking BMI ≥ 30

• Previous adverse pregnancy outcome suggestive of undiagnosed GDM e.g. shoulder dystocia, unexplained stillbirth

• Previous baby with birth weight > 4.5kg

• Previous GDM

• Strong Family History Diabetes (e.g. first degree relative with diabetes; or sister with GDM).

An OGTT should be performed in early pregnancy (ideally ≥ 13 weeks gestation) in a woman with any of the above risk factors. If the test is negative, a 75g 2-hour OGTT should be repeated at 24-28 weeks gestation^36^.
