# Supplementary material for: Development and evaluation of an online questionnaire to identify women at high and low risk of developing gestational diabetes mellitus
Source: BMC Pregnancy Childbirth. 2022 Apr 14;22:321. doi: 10.1186/s12884-022-04629-8 (PMC9009497; doi:10.1186/s12884-022-04629-8)
Supplement: Supplementary file 6 — Additional file 6. Supplementary file 6. [file 12884_2022_4629_MOESM6_ESM.pptx]

## Slide 1
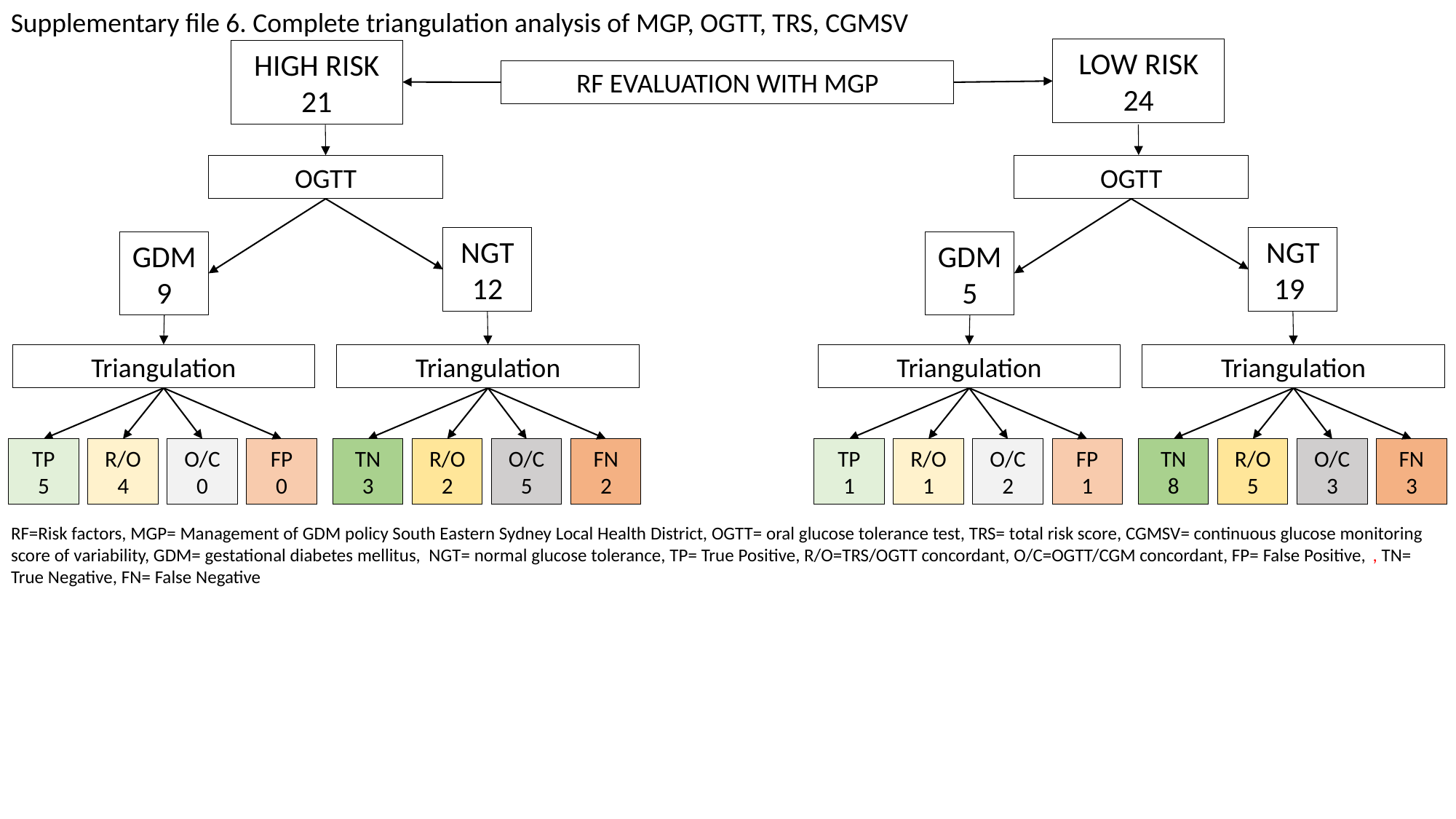

Supplementary file 6. Complete triangulation analysis of MGP, OGTT, TRS, CGMSV
LOW RISK
24
HIGH RISK
21
RF EVALUATION WITH MGP
OGTT
OGTT
NGT
12
NGT
19
GDM
9
GDM
5
Triangulation
Triangulation
Triangulation
Triangulation
TP
5
R/O
4
O/C
0
FP
0
TN
3
R/O
2
O/C
5
FN
2
TP
1
R/O
1
O/C
2
FP
1
TN
8
R/O
5
O/C
3
FN
3
RF=Risk factors, MGP= Management of GDM policy South Eastern Sydney Local Health District, OGTT= oral glucose tolerance test, TRS= total risk score, CGMSV= continuous glucose monitoring score of variability, GDM= gestational diabetes mellitus, NGT= normal glucose tolerance, TP= True Positive, R/O=TRS/OGTT concordant, O/C=OGTT/CGM concordant, FP= False Positive, , TN= True Negative, FN= False Negative
